# ISG15 modulates inflammatory profiles and ability to activate CD8 + T cells in bone marrow-derived dendritic cells

**DOI:** 10.1007/s00018-025-05849-9

**Published:** 2025-10-24

**Authors:** Pedro Martínez-Fleta, Clara Pertusa, Irene Fernández-Delgado, Raúl Izquierdo-Serrano, Marta Ramírez-Huesca, Nieves Fernández-Gallego, Diego Calzada-Fraile, Elena Moya-Ruiz, Raquel Castillo-González, Susana Guerra, Enrique Martín-Gayo, Francisco Sánchez-Madrid

**Affiliations:** 1 Universidad Autónoma de Madrid, Madrid, Spain; 2 Department of Medicine, Universidad Autónoma de Madrid, Madrid, Spain; 3 Vascular Pathophysiology Area, Centro Nacional Investigaciones Cardiovasculares (CNIC) Carlos III, Madrid, Spain; 4 Escuela de Doctorado, Universidad Autónoma de Madrid, Madrid, Spain; 5 Department of Immunology, Ophthalmology and Ear, Nose and Throat (ENT), Complutense University School of Medicine and Instituto de Investigación Sanitaria Hospital 12 de Octubre (imas12), Madrid, Spain; 6 Departamento de Medicina Preventiva y Salud Pública y Microbiología, Universidad Autónoma de Madrid, Madrid, Spain; 7 Centro de Biología Molecular Severo Ochoa, CSIC, Madrid, Spain; 8 CIBER Infectious Diseases (CIBERINFEC) from Instituto de Salud Carlos III, Madrid, Spain; 9 CIBER Cardiovascular CIBERCV, Madrid, Spain

**Keywords:** ISG15, Dendritic cells, Inflammasome, IL-1β

## Abstract

The Interferon-Stimulated Gene 15 (ISG15) has a key role during viral infections, since it can modify and regulate the expression of proteins from the host and from viruses. *Isg15*^−/−^ mice are more susceptible to infectious diseases. ISG15 is induced by type I Interferons (IFN-I) and apart from defense against pathogens, it can also play an important function in cancer or immune-mediated inflammatory diseases. Previous studies reported that ISG15 expression is increased in post-synaptic dendritic cells (DCs) upon interaction with CD4 + T cells. However, data regarding the role of ISG15 in DCs are scarce. The present study assesses the function of ISG15 in DCs activation, migration and ability to mediate T cell activation using bone marrow-derived DCs (BMDCs) and stimulation with lipopolysaccharide (LPS). Activation of DCs was not impaired but tended to be lower in *Isg15*-deficient mice, as observed by reduced CD40 induction in *Isg15*^−/−^ BMDCs. *Isg15*^−/−^ BMDCs induced less proliferation and Granzyme B expression in co-cultured CD8 + T cells. Interestingly, *Isg15*^−/−^ BMDCs secreted reduced levels of the pro-inflammatory cytokines IL-1β and IL-12 upon LPS stimulation. Mechanistically, our data suggest that ISG15 regulates Caspase-1 activity in DCs leading to lower IL-1 β secretion. In conclusion, this study reveals an essential role for ISG15 modulating DCs inflammatory activity and raises new questions regarding the specific mechanisms of how this protein regulates innate immune responses.

**Supplementary Information:**

The online version contains supplementary material available at 10.1007/s00018-025-05849-9.

## Introduction

Post-translational modifications (PTMs) are one of the most important and functionally diverse regulatory systems of protein location, stability, and function. PTMs include a wide group of modifications, e.g. phosphorylation, acetylation, etc. An essential PTM is elicited by the conjugation of small proteins. Ubiquitin (UB) and Ubiquitin-like-modifiers (UBLs) are included in this group and characterized by a β-grasp fold and the ability to covalently modify proteins mediated by a conjugation enzyme machinery [1, 2]. Interferon-Stimulated Gene 15 (ISG15) is an inducible modifier protein expressed mainly in response to type I IFNs and Pathogens Associated Molecular Patterns (PAMPs) such as bacterial lipopolysaccharide (LPS), viral double-stranded RNA (dsRNA), single-stranded DNA (ssDNA), vascular endothelial growth factor (VEGF), tumor necrosis factor-α (TNF-α), retinoic acid or certain genotoxic stressors. Some of them induce ISG15 by triggering a type I IFN response (retinoic acid or LPS) whereas other PAMPs, such as dsDNA and dsRNA, can induce it in a type I IFN-independent manner [3]. Its conjugation to proteins is controlled by the sequential action of three enzymes: activating E1 (Ube1L), conjugating E2 (UbCM8) and E3 ligase (HERC6). ISG15 can interact with or modify a wide variety of protein targets, modulating their function in diverse ways, in processes such as viral infection, extracellular vesicles (EVs) secretion, immune modulation, autophagy or tumorigenesis [4].

One of the main functions of ISG15 is the defense against viral infections. Specifically, ISG15-deficient mice are more susceptible to influenza, herpes simplex, murine gammaherpervirus or Sindbi virus [5]. ISG15 hampers viral replication via conjugation to viral proteins, marking them for degradation or impeding their function [6–8]. ISG15 can act as a PTM or as a free extracellular molecule. The integrin lymphocyte function associated antigen 1 (LFA-1) is a putative receptor for free extracellular ISG15 [9]. This integrin is responsible for leukocyte migration through binding of ligands such as intercellular adhesion molecules (ICAM) [10]. Thus, ISG15 may also modulate cell migration through its interaction with LFA-1 [11]. ISG15 can be secreted by different cell types, including lymphocytes and epithelial cells, thereby modulating antiviral response of NK and T cells by inducing IFN-γ secretion [12]. Extracellular ISG15 mediates activation of CD8 + T cells [13] and is important for IFN-γ secretion in CD4 + and CD8 + T cells [14]. Regarding innate immune cells, dendritic cells (DCs) are key players in mounting a proper immune response against all kind of pathogens. Apart from antigen presentation, DCs also play a role in activation of different immune cells through secretion of several cytokines [15]. During infection, PAMPs are recognized by Toll Like Receptors (TLRs) expressed in DCs [16]. These PAMPs can activate different signaling pathways, such as NF-kB, which leads to transcription of pro-inflammatory cytokines like IL-1β. Prior to IL-1β secretion, the proIL-1β has to be cleaved by Caspase-1. Caspase-1 needs to be activated by a multiprotein complex, the NLRP3 inflammasome. The action of NLRP3 inflammasome, together with Caspase-1 leads to the cleavage of proIL-1β and proIL-18 that allows secretion of these pro-inflammatory cytokines [17]. Furthermore, ISG15 induction after TLR priming could be relevant for NLRP3 inflammasome stability and activity [18].

Several studies have been focused on deciphering the function of ISG15 in regulating T and NK cell activation, or in unveiling its role in homeostasis and how it induces a coordinated immune response in mouse models. Previous studies have revealed significant transcriptomic changes in post-synaptic DCs (psDCs), including the upregulation of *Isg15*, suggesting that it could participate in the functional specialization of psDC [19]. In addition, extracellular ISG15 has been reported to increase the production of IL-1β in CD8α + dendritic cells (DCs) in a *Toxoplasma gondii* infection model in mice [20]. However, mechanisms by which ISG15 could regulate caspase-1 or inflammasome activity and its potential link with functional properties of DC has not been studied in detail. The present study aims to shed light on the functional role of this protein modifier in this innate immune cell lineage. Results presented here indicate that ISG15 can modulate maturation and activation of BMDCs and the lack of this protein modifier in these cells negatively affects their ability to mediate proliferation and activation of CD8 + T cells. In addition, ISG15 is necessary for the release of pro-inflammatory cytokines such as IL-1β and IL-12 by BMDCs. Our data suggest that ISG15 may regulate Caspase-1 protein necessary for pro-IL1-β cleavage and secretion. These results unveil novel functions of ISG15 in BMDCs that may account for their activity and inflammatory profiles.

## Methods

### Mice

*Isg15*^*−*/−^ mice (C57BL/6 background), also referred to as KO or ISG15-KO, were kindly provided by Klaus-Peter Knobeloch laboratory [21]. For immune synapse (IS) experiments TCR (Vα2, Vβ5) transgenic mice (OT-II), and TCR (Vα2, Vβ5) transgenic mice (OT-I) were used [19]. Experiments were not blinded, as the experimental groups (WT and KO) were previously identifiable by genotype. Animals were randomly assigned to experimental groups when applicable. All experiments were performed using male and female mice of 8–12 weeks kept on a regular 12 h light/dark cycle (7 a.m. − 7 p.m. light period), with food and water available *ad libitum*. All mice were bred and maintained in the pathogen–free animal facilities of the Centro Nacional de Investigaciones Cardiovasculares (CNIC, Madrid, Spain) under the animal care standards of the institution. All experimental procedures with animals were approved by Institutional Animal Care Committee following Spanish and European guidelines (Proex 210, 201.6, 206.1).

### Antibodies (Abs) and other reagents

Abs and reagents used are listed in Supplementary Table 1.

### Generation of bone marrow-derived dendritic cells (BMDCs)

Bone marrow (BM) cell suspensions were extracted from medullary cavity of femur and tibia from both WT and ISG15-KO mice. ACK lysis buffer (Lonza, 10-548E) was used to lysate erythrocytes for 30 s at room temperature (RT) and cell suspensions were filtered through a 70-µm cell sieve (Fisher Scientific, 10788201). BM cell suspension was cultured on non-treated 150-mm Petri dishes at a concentration of 5·10^5^ cells/mL in complete RPMI-1640 supplemented with 20 ng/mL of recombinant mouse Granulocyte-Macrophage Colony Stimulating Factor (GM-CSF, PeproTech). Adhered cells were removed from culture at day 3. Cells (floating + attached) were maintained and passed every two days by detachment with PBS 1x– 5% BSA– 5 mM EDTA (PBE). GM-CSF BMDCs were collected at day 9 for experiment and characterized as CD11c+ Gr-1− cells by flow cytometry (Supplementary Fig. 1).

### Flow cytometry (FC)

Cultured BMDCs were stained with LIVE/DEAD^®^ Fixable Yellow Dead Cell Stain (Invitrogen) and a combination of monoclonal antibodies directed to CD40, CD8 (BioLegend), MHC-II (Invitrogen), CD4, CD86, CD11c, Ly6G (GR-1), IFN-γ (Tonbo Biosciences), IL-1β (Invitrogen), IL-12 (BD Biosciences), Granzyme B (eBioscience). For intracellular staining of cytokines, cells were cultured in the presence of 2.5 µg/mL of Brefeldin A (Sigma-Aldrich) and Monensin (Sigma-Aldrich) that were added 2 h after LPS treatment and left overnight (O/N) until cells were collected for the staining.

Cell suspensions were transferred to a 96-V-well plate (BRAND™) and incubated in LIVE/DEAD^®^ Fixable Yellow Dead Cell Stain (Invitrogen), FcBlock (Tonbo), 5 mM EDTA and PBS 1x for 20 min at 4 °C. Solution was washed out with PBE. Cells were incubated with surface primary Ab cocktail (for dilution see Supplementary Table 1) for 1 h at 4 °C. Solution was washed out with PBE. For fixation, 1% Paraformaldehyde (PFA) in 5 mM EDTA - PBS 1x was used for 15 min at 4 °C. When intracellular antigens (Ags) were targeted, after washing out surface primary Ab cocktail, cells were incubated with Cytofix/Cytoperm™ (BD) for 20 min at 4 °C. Solution was washed out with Perm/Wash™ Buffer (BD). Then, cells were incubated with intracellular primary Ab cocktail in Perm/Wash™ Buffer for 1–2 h at 4 °C. Alternatively, for intranuclear staining, after washing out surface primary Ab cocktail, cells were incubated with FoxP3 Fixation/Permeabilization staining Buffer (eBioscience, 00-5523) for 20 min at 4 °C. Solution was washed out with FoxP3 Permeabilization staining Buffer (eBioscience, 00-8333). Then, cells were incubated with intranuclear or intracellular primary Ab cocktail in Permeabilization Buffer for 1 h at 4 °C. Cells were analysed in FACS CantoTM II, LSRFortessaTM or FACS SymphonyTM. As single positive controls UltraComp eBeads (Invitrogen, 01-2222-41) or cells were used.

### Reverse transcription and real-time quantitative PCR (qPCR)

Total RNA (0.5 to 2 µg) was reverse transcribed to cDNA with High-Capacity cDNA Reverse Transcription Kit with RNase Inhibitor (Applied Biosystems, 4374966). Then, qPCR was performed using GoTaq^®^ qPCR Master Mix (SYBR Green, Promega, A6001) in an AB7900-384 thermocycler (Applied Biosystems). Dilutions were performed to cDNA when needed, ranging from 15 to 100 ng per well. PCR reactions were performed by triplicate in 384-well plates. Expression levels of target genes were normalized to housekeeping genes *Ubc* and *Hprt*. Gene-specific primers used are listed in Supplementary Table 2.

### Enzyme-Linked Immunosorbent Assay (ELISA)

Cytokine production was analysed in the supernatant of GM-CSF BMDCs treated with LPS for 12–24 h at 37 °C. Dilutions were performed to maintain measures in standard curve range. Detection was based on colorimetric quantification of absorbance at 450 nm, corrected by subtraction at 570 nm in a microplate reader (Bio-Rad 550) using MaxiSorp^®^ flat-bottom 96 well plates (Nunc). Cytokines analysed are described in Supplementary Table 3.

### Western blot

A total of 1.5 × 10^6^ cells from ISG15-KO and WT mice were lysed in WB lysis buffer (TBS with 1% NP40, 0.5% sodium deoxycholate, 0.1% SDS) supplemented with protease inhibitors (Complete, Roche) and phosphatase inhibitors (PhosSTOP, Roche). Total protein was quantified by a Pierce™ BCA protein assay kit (Thermo Scientific) and 30 µg of total protein per lane were loaded. Proteins were separated by SDS-PAGE in non-reducing or reducing conditions on 8–12% acrylamide/bisacrylamide (29:1, 30%, BioRad) gels and transferred to a nitrocellulose or PVDF membrane (200 mA for 120 min at 4 °C). Membranes were blocked with 5% BSA-TBS for 1 h, incubated with primary Abs O/N at 4 °C (for dilution see Supplementary Table 1) and peroxidase-conjugated secondary Abs (1:5000) for 40 min at RT. Proteins were visualized with Immobilon (Millipore, WBLUF0500) using ImageQuant LAS-4000 Mini (GE Healthcare) or iBright 1500 (Invitrogen).

### In vitro migration assay (Transwell)

GM-CSF BMDCs were cultured on top of 5 μm pore size polycarbonate membrane (Costar#3421) in 100 µL of 0.1% BSA-RPMI-1640, over 600 µL of the same medium (basal) or containing mouse CCL21 (100 ng/mL). A well was filled with the same number of cells without the membrane as input. Cells were let to migrate for 2–3 h at 37 °C. Afterwards, migrated cells were recovered from the lower compartment and analyzed by flow cytometry; input cells were used as controls. Cells were stained with a viability dye (7AAD) and CD11c Ab and migrated cells were counted and normalized using BD Trucount Absolut Counting Tubes IVD (BD).

### BMDCs and T cell co-culture

GM-CSF BMDCs were treated with LPS (250 ng/mL) O/N at 37 °C for DC maturation. Next day, LPS was withdrawn and DCs were pulsed with the corresponding peptide (Supplementary Table 1) for at least 2 h. Then, DCs were cocultured for 24 h with OT-II purified CD4 + or OT-I purified CD8 + T cells (DCs: T cell ratio 1:2). For ovalbumin (OVA) protein processing and Ag presentation, DCs were pulsed with whole OVA protein simultaneously with LPS and withdrawn the day after. T cell proliferation was assessed using CellTrace Violet and T cell activation was determined by using IFN-γ and Granzyme B monoclonal antibodies by Flow Cytometry.

### Treatment of BMDCs with rISG15

BMDCs were cultured in the presence or not of 1 µg/mL LPS, 20 ng/mL rISG15 and/or 20 ng/mL rIL-12 and cells and supernatants were collected 12 and 24 h after addition of stimuli. For extracellular ISG15 blocking experiments, BMDCs were cultured with or without 1 µg/mL LPS and 200 ng/mL of ISG15 polyclonal antibody (Cell Signaling) or isotype control (BioLegend) and left 24 h after collection of supernatants.

### Skin painting

For DC migration experiments in vivo, 10 µL of FITC 1% dissolved in Acetone: Dibutyl-phyalate (1:1) were administered in mice ears for sensitization. FITC 1% dissolved in acetone was used as vehicle control. Mice were sacrificed 48 h after administration and auricular LN (aLN) were processed and analysed for DC migration by FC.

### Statistical analysis

Data were analyzed with GraphPad Prism software v.9.0 (La Jolla, CA). Normality was studied with D’Agostino-Pearson omnibus or Shapiro-Wilk normality test. When data passed the normality test (α > 0.05), a parametric test was applied, Student’s *t*-test for two groups or one-way analysis of variance (ANOVA) test with Tukey’s post-test for multiple comparisons when including more than two groups. When working with dependent samples, a paired *t* test was conducted. For nonparametric data, Mann-Whitney *U* test or Kruskal-Wallis with Dunn’s post-test were applied for two or more groups, respectively. Every experiment was analyzed through at least three biological replicates. Graphs show the distribution of each sample and mean ± standard deviation (SD). Tukey-style Box and whiskers plot represents median, lower quartile, upper quartile and two independent whiskers allowing outliers. Statistically significant differences were considered when *p* ≤ 0.05 (depicted as *), *p* ≤ 0.01 (**), *p* ≤ 0.001 (***) and *p* ≤ 0.0001 (****). Not significant differences were represented as ns (*p* > 0.05).

## Results

### TLR agonists induce high levels of ISG15 in BMDCs

**Fig. 1:**
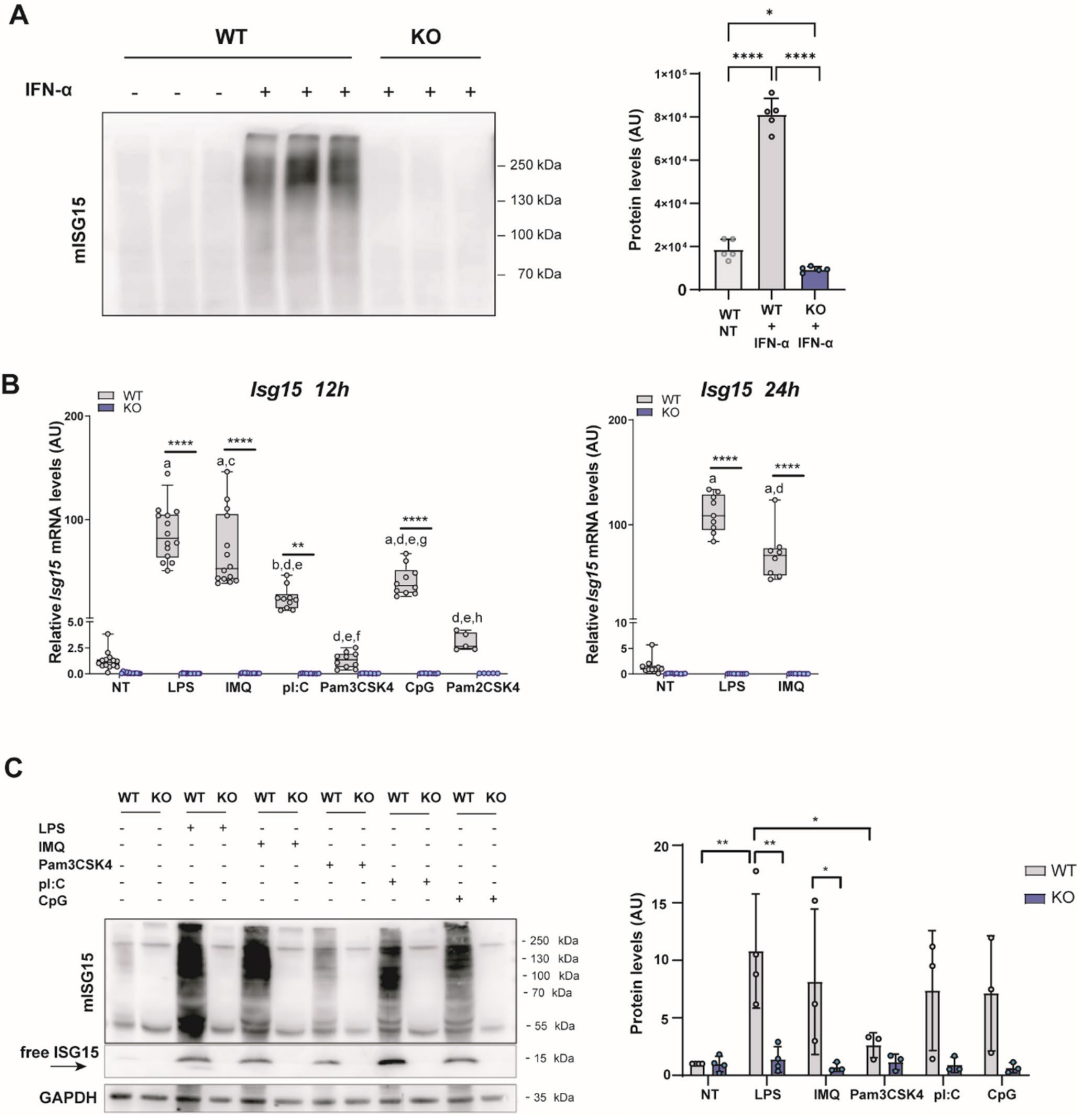
ISG15 induction upon treatment with different PAMPs in murine BMDCs. **A** ISG15 protein levels assessed by Western blot in BMDCs from WT and ISG15-KO mice treated for 24 h with IFN-α. A total of 30 µg of total protein were loaded per lane. A representative gel with 3 biological replicates (left), along with the quantification of ISGylated proteins expressed in arbitrary units (right), *n* = 5; NT: non-treated. **B** *Isg15* mRNA expression levels measured by RT-qPCR in GM-CSF-derived BMDCs treated with different TLR agonists for 12 h (left panel) and 24 h (right panel). Relative expression was normalized to *Ywhaz* and *β-actin*. Data are combined from 3 independent experiments. Lipopolysaccharide (LPS), Imiquimod (IMQ), polyI: C (pI: C), Palmitoyl-2-CysSerLys-4 (Pam2CSK4), Palmitoyl-3-CysSerLys-4 (Pam3CSK4), CpG-oligodeoxynucleotides (CpG). **C** On the left, representative Western blot of BMDCs from WT and ISG15-KO mice treated O/N with various TLR ligands, showing ISGylated proteins and free-ISG15. On the right, quantification of total ISG15 normalized to GAPDH is shown. **A** *n*= 5, one-way-ANOVA with Tukey’s correction * *p* < 0.05, ****p < 0.0001. **B**-**C** Two-way ANOVA with Tukey’s correction (*n* ≥ 5 for B, *n* ≥ 3 for C). **B** **p ≤ 0.01 [vs KO], ****p ≤ 0.0001 [vs KO], a: *p* ≤ 0.0001 [vs NT], b: *p* ≤ 0.01 [vs NT], c: *p* ≤ 0.05 [vs LPS], d: *p* ≤ 0.0001 [vs LPS], e: *p* ≤ 0.0001 [vs IMQ], f: *p* ≤ 0.01 [vs pI: C], g: *p* ≤ 0.0001 [vs Pam3CSK4], h: *p* ≤ 0.0001 [vs CpG]. **C** *p < 0.05, **p ≤ 0.01.

BMDCs from *Isg15*^*−*/−^ mice were generated and lack of expression of mISG15 was confirmed by Western blot analysis prior to assess the role of ISG15 in BMDCs. Protein ISGylation was induced by IFN-α in BMDCs from WT but not in cells from ISG15-KO mice (Fig. 1A). Different primary DC subtypes display specific TLR pattern expression and responsiveness depending on their origin and function [22–24]. Therefore, we performed a detailed study of ISG15 inducible expression (and ISGylation) on BMDCs by different TLR agonists, from extracellular ligands such as LPS (TLR4), Pam_3_CSK_4_ (TLR2/1), Pam_2_CSK_4_ (TLR2/6); to intracellular TLR ligands such as pI: C (TLR3), IMQ (TLR7/8) and CpG (TLR9). Consistent with the reported function of ISG15 in antiviral responses, we observed high *Isg15* mRNA expression when cells were pulsed with TLR stimuli based on DNA/RNA, but also at significant high levels upon LPS challenge after 12 and 24 h of stimulation (Fig. 1B**).** Consistently, higher levels of protein ISGylation, as well as free intracellular ISG15, upon these TLR-agonist treatments was also observed in WT but not in *Isg15*^*−*/−^ mice (Fig. 1C). Therefore, these data show that intracellular ISG15 is induced after stimulation with IFN-α in BMDCs and that LPS can induce high levels of ISG15 compared to other TLR agonists.

### Ability of BMDCs to mediate CD8 + T cell proliferation is dependent on ISG15

**Fig. 2:**
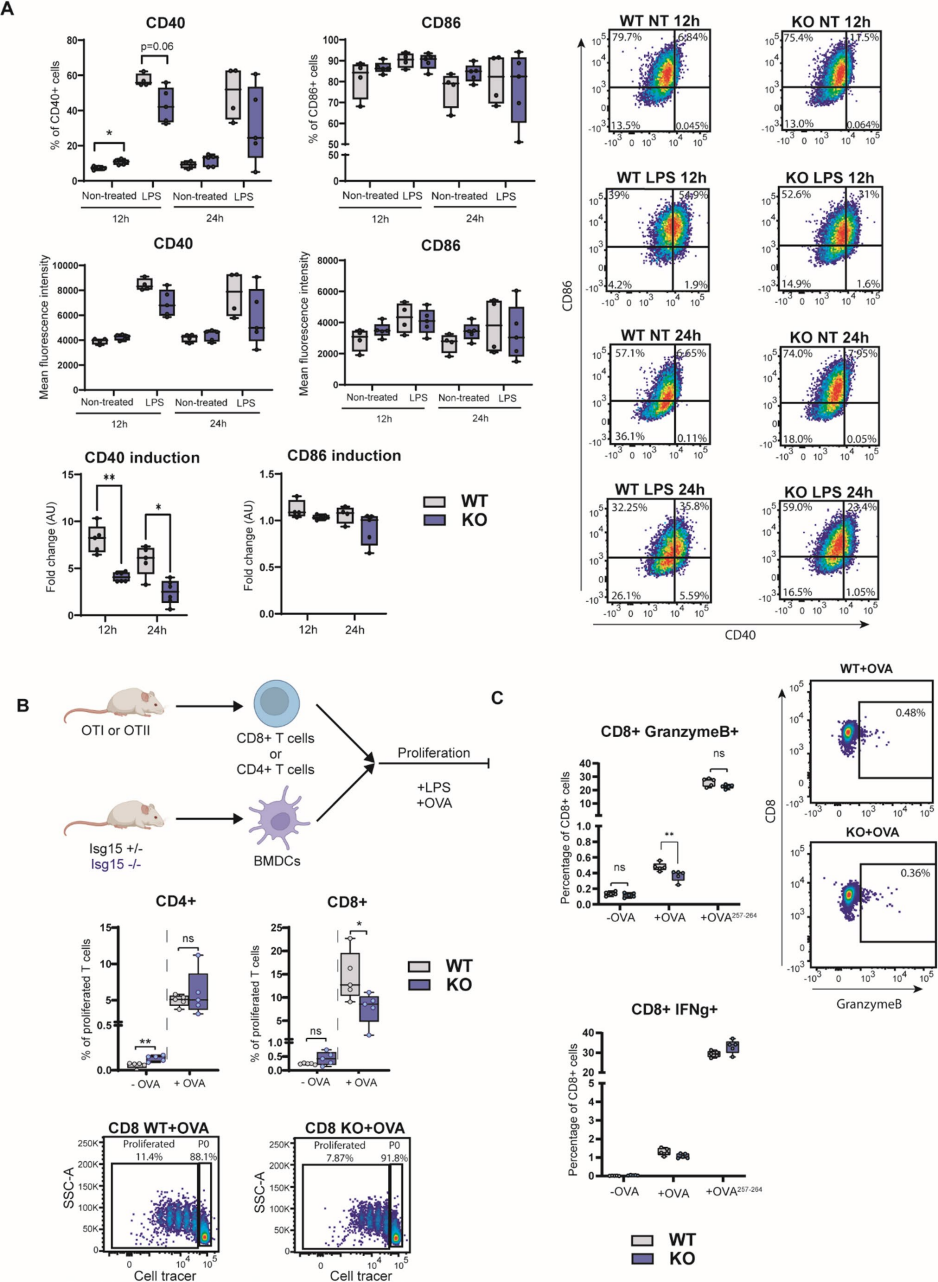
Activation, proliferation and antigen presentation capacity of ISG15-KO BMDCs. **A** Expression of CD40 and CD86 in BMDCs shown as percentage of positive cells (top), mean fluorescence intensity (MFI, middle) and fold change induction (bottom). Representative flow cytometry dot plots are included. Data are representative of 3 independent experiments (*n* ≥ 4). NT: non-treated. **B** Schematic of the experimental workflow for BMDC-T cell coculture (top). T cells were labelled with CellTrace prior to stimulation and decreased CellTrace Violet signal was interpreted as proliferation (*n* = 5 biological replicates). Frequencies of proliferating CD4 + T cells from OT-II mice and CD8 + T cells from OT-I mice after 72 h of co-culture with BMDCs from WT or ISG15-KO mice, with or without treatment with OVA protein (middle) and representative flow cytometry plots showing CellTrace Violet dilution (bottom). **C** Frequencies of naïve CD8 + T cells from OT-I mice expressing Granzyme B and IFN-γ 24 h after co-culture with BMDCs from WT or ISG15-KO mice, in the presence or absence of OVA protein or OVA_257−264_ peptide (*n* = 5 biological replicates). A viability dye was used for live-cell gating and unstained controls were included to define positive populations. **A-C** Mann-Whitney test (*n* = 5) *p < 0.05, **p < 0.01; ns, not significant

Next, since LPS induced the highest levels of ISG15, we assessed the activation and maturation status of ISG15-KO BMDC cells in response to this PAMP. We monitored the surface membrane levels of the activation markers CD40 and CD86 on BMDC from WT and KO mice stimulated with LPS (Fig. 2A, Supplementary Fig. 2A). Our results showed that ISG15-KO BMDCs expressed slightly but significantly higher proportions of CD40 + cells at baseline, indicating a potential higher basal activated state than WT DCs (Fig. 2A, top). Although ISG15-KO BMDCs were capable of increasing CD40 expression upon LPS stimulation, we observed a significant decrease of the ability to induce CD40 in cells from KO compared to WT mice, suggesting less effective induction of cell maturation (Fig. 2A, bottom). No differences in the percentage of BMDCs expressing CD86 after LPS treatment were observed, although as for CD40, induction ability of CD86 was slightly decreased, although non-significant (Fig. 2A).

After its arrival at a draining lymph node (dLN), a crucial step for a DC is antigen (Ag) presentation through an intimate contact with a T cell at the IS. Hence, we wondered whether ISG15 may have a role in BMDC-mediated T cell activation. To address that, we differentiated BMDCs from *Isg15*-WT or KO mice, pulsed them with whole ovalbumin (OVA) protein and co-cultured with naïve CD8+ T cells or naïve CD4+ T cells, isolated from the OVA Ag-specific mouse model OT-I or OT-II, respectively (Fig. 2B, top). Interestingly, we observed a significant reduction in proliferation of CD8 + T cells, but not CD4 + after 72 h upon activation by BMDCs from *Isg15-*deficient mice (Fig. 2B middle). Furthermore, CD8 + T cells primed with ISG15-KO BMDCs presented lower Granzyme B expression than those primed with WT BMDCs (Fig. 2C top, Supplementary Fig. 2B), thus suggesting a defective activation of cytotoxic CD8 + T cells. However, IFN-γ + CD8 T cells remained unchanged (Fig. 2C bottom). Therefore, ISG15-KO BMDCs display reduced abilities to mediate proliferation and activation of cytotoxic CD8 + T cells.

We then considered the possibility that migration may be affected in ISG15-KO BMDCs. To test this hypothesis, we assessed in vitro the migratory capacity of ISG15-KO BMDCs upon chemoattraction in response to CCL21, a ligand of CCR7 highly expressed in activated DC, by performing a transwell assay. We observed that KO BMDCs preserved their ability to migrate in response to CCL21, as similar chemoattraction migration activities were observed upon LPS-induced maturation in both ISG15-KO and WT BMDCs (Supplementary Fig. 3A). Moreover, we also assessed the global migratory capacities in vivo. To do that, mice were challenged with an inflammatory stimulus (Dibutylphtalate) in combination with FITC. The analysis of FITC + migratory DCs in aLNs revealed no differences in migratory capacities towards LNs between KO and WT cells (Supplementary Fig. 3B). Together, these results indicate that ISG15 mediates maturation of DCs, as well as activation and proliferation of CD8 + T cells, but not DC migration.

### Reduced secretion of IL-1β in ISG15-deficient BMDCs primed with LPS

**Fig. 3:**
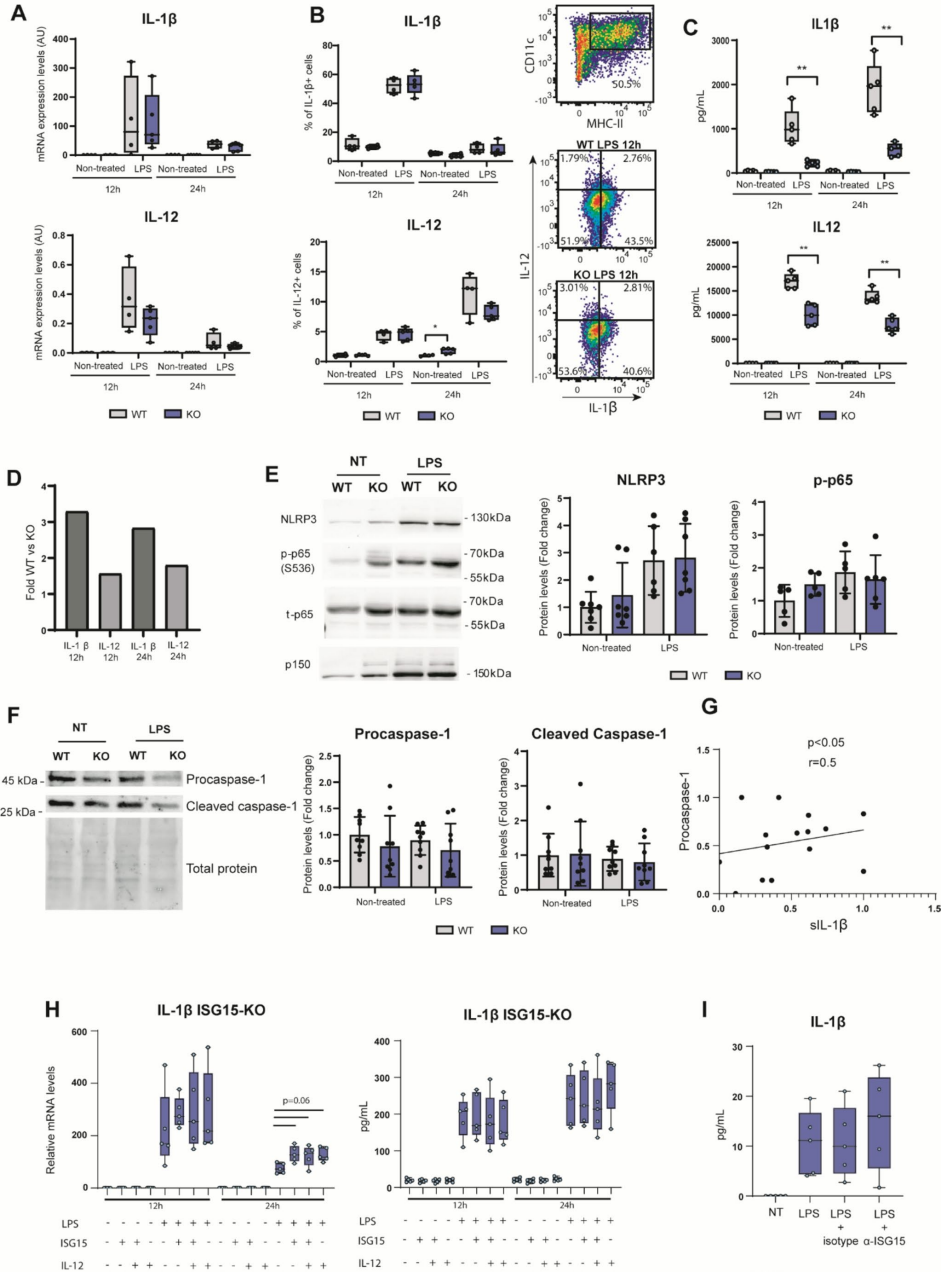
Expression of IL-1β and IL-12 and inflammasome activity in ISG15-KO BMDCs. **A** *Il-1β* and *Il-12* mRNA expression levels assessed by RT-qPCR in BMDCs, normalized to *Hprt* and *Ubc*. **B** Intracellular cytokine staining and representative flow cytometry gatings. Fluorescence minus one (FMO) control samples were used to define positive populations. **C** Secreted protein measured by ELISA. **A**, **B**, **C** *n* = 5, representative data of 3 independent experiments. **D** Reduction of secreted IL-1β and IL-12 levels in KO vs. WT BMDCs, shown as fold change of medians. **E** Representative Western blot of NLRP3, p-p65, total p65 in BMDCs from WT and ISG15-KO mice. p150 was used as loading control. Bar plots show fold change relative to non-treated (NT) WT and normalized to p150 (*n* = 7, two independent experiments). **F** Representative Western blot showing protein expression of procaspase-1 and its cleaved form in WT and ISG15-KO BMDC. Total protein was used as loading control. Quantification is shown as fold change relative to non-treated WT, normalized to total protein (*n* = 9, two independent experiments). **G** Correlation between levels of soluble IL-1β (sIL-1β) and procaspase-1 in BMDCs from WT and ISG15-KO mice after LPS stimulation. Values of both variables were normalized and are shown as arbitrary units (*n* = 15, Spearman correlation). **H** *Il-1β* mRNA levels (left) and IL-1β protein secretion measured by ELISA (right) in ISG15-KO BMDCs, after treatment with LPS and exogenous addition of 20 ng/mL of rISG15 and/or rIL-12. **I** Levels of extracellular IL-1β were assessed upon treatment with LPS and blockage of extracellular ISG15 using polyclonal anti-ISG15 antibody in WT BMDCs. NT: non-treated (H)(I) data were normalized by cell count. *n* = 5, data are representative of 3 independent experiments. Data in (**A-F**, **H-I**) were analyzed using the Mann-Whitney test (*n* = 5 unless otherwise stated). *p < 0.05, **p < 0.01, and ***p < 0.001

DCs have the ability to produce key inflammatory cytokines that will modulate and determine polarization of T cells and hence, the fate of adaptive immune responses [25, 26]. Therefore, we addressed the role of ISG15 in expression and secretion of proinflammatory cytokines downstream TLR after LPS stimulation such as IL-1β and IL-12 in BMDCs. Our data showed no differences in levels of *Il-1β* and *Il-12* mRNA in ISG15-KO BMDCs compared to WT after 12 h and 24 h of treatment with LPS (Fig. 3A). We also assessed the levels of these intracellular cytokines by a flow cytometry intracellular staining (Supplementary Fig. 4), resulting in similar expression between WT and KO after LPS treatment for both IL-1β and IL-12, although a slight decrease was observed in proportions of IL-1β + cells in non-treated ISG15-KO BMDCs after 24 h of culture (Fig. 3B). Interestingly, after measuring extracellular levels of secreted IL-1β and IL-12 in BMDC cultures, we observed a clear reduction of these cytokines in the supernatant of ISG15-KO BMDCs after 12 h and 24 h of LPS treatment **(**Fig. 3C**)**. Moreover, ISG15-KO BMDCs showed a higher reduction in secretion of IL-1β than IL-12 upon LPS stimulation (Fig. 3D).

In order to rule out a defect in NF-кB pathway or NLRP3 inflammasome, we also determined levels of p65 phosphorylation and total levels of NLRP3. However, no significant reduction in detection of these proteins was observed upon LPS stimulation in KO vs. WT BMDCs (Fig. 3E). Therefore, we then interrogated the possibility that ISG15-deficient cells may have a defect in IL-1β cleavage and maturation. We assessed levels of Caspase-1 in ISG15-KO BMDCs. Our results show a trend to lower levels of procaspase-1 in ISG15-KO BMDCs (Fig. 3F). This reduction does not seem to be a result of enhanced cleavage into its active form but reduced levels of the procaspase itself (Fig. 3F). Moreover, procaspase-1 levels positively correlated with soluble IL-1β levels (Fig. 3G), further supporting that expression of procaspase-1 is required for efficient IL-1β secretion. In addition, mRNA levels of *Caspase-1* and other genes involved in different inflammasomes such as *Nlrp3*, *Aim2* or *Ifi204* were also analysed by RT-qPCR, revealing no significant differences between WT and KO BMDCs (Supplementary Fig. 5). Furthermore, based on previous observations [9], we wondered which could be the role of free extracellular recombinant ISG15 (rISG15) in regulating levels of IL-1β secretion. We evaluated the levels of both mRNA *Il-1β* and extracellular IL-1β in *Isg15*-deficient BMDCs after addition of rISG15 and IL-12 to cell culture and upon 12 h and 24 h of LPS treatment. These data show an increase in mRNA levels of *Il-1β* upon rISG15 treatment. However, extracellular levels of IL-1β remained unchanged after the addition of rISG15 in KO BMDCs **(**Fig. 3H**)**. Finally, blockage of free extracellular ISG15 by addition of polyclonal anti-ISG15 antibodies did not significantly change secreted amounts of IL-1β by WT BMDCs **(**Fig. 3I**)**. Thus, our results suggest that intracellular ISG15 regulates procaspase-1 expression, required for effective processing of active IL-1β after LPS stimulation.

## Discussion

In this study, we have addressed the role of ISG15 in BMDCs upon activation with the TLR4 ligand LPS. We assessed the effect of this interferon-induced protein on BMDC activation, cytokine production and secretion, migration, and capacity of priming T cells. Our results show that activation of *Isg15*^*−/−*^ BMDCs is not severely impeded, although induction of the activation marker CD40 was significantly decreased.

ISG15 (mRNA and protein) was induced at higher levels by LPS and TLR ligands based on DNA/RNA in BMDCs. This is consistent with other works in which secretion of ISG15 in PBMCs was higher after stimulation with heat killed *Salmonella typhimurium* and poly I: C [12]. ISG15-deficient BMDCs seem to be more activated basally but they do not reach a complete activation upon LPS treatment, as observed after measuring CD40 levels in DCs. CD40 is efficiently induced by type I IFN responses and promotes in turn the upregulation of type I IFN, a process that is enhanced in differentiated DCs. Consequently, since ISG15 is involved in type I IFN signaling, this protein could be regulating CD40 expression in BMDCs [27, 28]. Our results suggest that ISG15 could also be modulating CD86, although its impact on CD86 expression was less clear. On the other hand, although we did not perform these experiments, free extracellular ISG15 might indeed regulate CD86 in DCs, as previously suggested [29].

Our experiments carried out to assess the effect of ISG15 on antigen presentation by DCs have revealed a reduction of CD8 + but not CD4 + T cell proliferation in ISG15-deficient BMDCs. Previous works have associated ISG15 with CD8 + T cell activation via NK cell activation and the subsequent cytokine secretion [13]. Interestingly, the decrease in CD40 expression in DCs could explain the reduced proliferation of CD8 + T cells due to the importance of CD40-CD40L interaction for T cell expansion, as previously described [30]. Inflammasome activation might be relevant to prime CD8 + T cell responses [31] and previous studies have shown that mice deficient in Caspase-1 or IL-1R induced lower CD8 + T cell activation and were less protected against influenza infection [32]. Therefore, Caspase-1 in DCs may be required for effective stimulation of CD8 + T cells.

ISG15 modulates IL-12 release, and this cytokine is important for Th1 differentiation, CD8+ T cell activation, cytotoxicity and NK cell activation [26, 33–35], We therefore hypothesize that the reduced secretion of IL-12 observed in ISG15-KO BMDCs might account for the decrease in CD8 + T cell proliferation and that this observation may not necessarily result in a defect in antigen presentation by these cells. Consistent with this, the reduced levels of Granzyme B in CD8 + T cells observed may be due to a deficient secretion of IL-12 by ISG15-KO BMDCs, since IL-12 has also been associated with increased expression of these cytotoxic proteins necessary for CD8 + T cell killing [36].

One of the main findings of this study is the reduced secretion of the pro-inflammatory cytokine IL-1β, as well as IL-12 in ISG15-KO BMDCs. Thus, ISG15 could be somehow regulating these pathways. Transcription and translation of both IL-1β and IL-12 were conserved in the absence of ISG15. However, the decrease in extracellular levels of these cytokines points towards the ISGylation as a key factor for IL-1β and IL-12 secretion. ISGylation may modulate directly these cytokines or other proteins that control them. In line with this, we searched for other proteins involved in IL-1β maturation and secretion. We identified a decrease in levels of Caspase-1 in ISG15-deficient BMDCs. This reduction in Caspase-1 might explain the decrease in IL-1β secretion whereas intracellular levels of IL-1β were unaltered in KO vs. WT DCs. On the other hand, free extracellular ISG15 has been reported to increase IL-1β secretion by CD8α + DCs (cDC1) at the site of infection [20]. Free extracellular ISG15 promotes the release of several cytokines in PBMCs. For instance, signaling through ISG15 induces IL-6, IL-1β and, in combination with IL-12, IFN-γ in PBMCs through binding to LFA-1 [9, 37–39]. To our knowledge, this is the first study that describes ISG15 as a key regulator of IL-1β release in BMDCs. Nevertheless, we were not able to restore IL-1β secretion by adding extracellular rISG15 to *Isg15*^-/-^ DCs, even in combination with IL-12. Additionally, antibody blockage of ISG15 did not alter secretion of IL-1β either. The fact that we did observe an increase in the transcription of *Il1β* after the addition of exogeneous ISG15 to ISG15-KO DCs, which is not accompanied by higher IL-1β secretion supports the possible role of intracellular ISG15 in regulating Caspase-1. However, whether other receptors that depend on the DC type may be mediating ISG15 signaling is a matter that merits further exploration. Moreover, GM-CSF-derived BMDCs are more similar to migratory CD8α- DCs (cDC2) [40], thus, different mechanisms and receptors may modulate ISG15 signaling.

We cannot rule out that ISG15 could also be regulating secretion of these cytokines by controlling multivesicular bodies formation and secretion, as previously reported [41]. In addition to IL-1β, secretion of IL-12 was also reduced in ISG15-KO. IL-12 and IL-1β have different release mechanisms. IL-1β depends mainly on Gasdermin D pores formation and plasma membrane permeabilization [42], and although some authors have reported that IL-1β can travel inside EVs [43], this is not the principal secretion mechanism. On the other hand, IL-12 is commonly found inside EVs [44]. This is a potential explanation why we also observed decreased IL-12 secretion from KO BMDCs, since ISG15 regulates EVs release [41]. The explanation why IL-1β decrease is more prominent than IL-12 in ISG15-KO DCs may rely on ISG15 effect on Caspase-1 stability. Further studies will be necessary to elucidate how ISG15 regulates inflammasome complex formation and whether it also participates in IL-12 release in these antigen-presenting cells. Our data support a novel role for ISG15 in DCs. Overall, this study provides evidence of the key functions of ISG15 in DC activation, pro-inflammatory cytokine secretion, and the promotion of CD8 + T cell expansion and activation. Additionally, although further experiments are needed to confirm the interaction between ISG15 and Caspase-1, we have characterized the role of ISG15 in IL-1β and IL-12 secretion by BMDCs. Thus, ISG15 ability to regulate key inflammatory molecules and adaptive immune responses makes it a promising target for the development of novel DC-based immunomodulatory therapies.

## Supplementary Information

Below is the link to the electronic supplementary material.

Supplementary Material 1 (DOCX 2.05 MB)

## Data Availability

All data relevant to the study are included in the article. Additional data are available upon reasonable request.

## Abbreviations

Abs: Antibodies
Ag: Antigen
aLN: Auricular lymph node
BM: Bone marrow
BMDCs: Bone marrow dendritic cells
dLNs: Draining lymph nodes
DC: Dendritic cell
dsRNA: Double-stranded RNA
EVs: Extracellular vesicles
GM-CSF: Granulocyte-macrophage colony-stimulating factor
ICAM: Intercellular adhesion molecule
IFN: Interferon
ISG15: Interferon stimulated gene 15
IL: Interleukin
IRF: Interferon regulatory factor
IS: Immune synapse
FC: Flow cytometry
LFA-1: Lymphocyte function associated antigen
LPS: Lipopolysaccharide
O/N: Overnight
OVA: Ovalbumin
PAMPs: Pathogen-associated molecular patterns
psDC: Post-synaptic dendritic cell
PTMs: Post-translational modifications
rISG15: Recombinant ISG15
RT: Room temperature
ssRNA: Single-stranded RNA
TLRs: Toll-like receptors
TNF-α: Tumor necrosis factor-α
UB: Ubiquitin
UBLs: Ubiquitin-like modifiers
VEGF: Vascular endothelial growth factor
