# Supplementary material for: ISG15 modulates inflammatory profiles and ability to activate CD8 + T cells in bone marrow-derived dendritic cells
Source: Cell Mol Life Sci. 2025 Oct 24;82(1):362. doi: 10.1007/s00018-025-05849-9 (PMC12552197; doi:10.1007/s00018-025-05849-9)
Supplement: Supplementary file 1 — Supplementary Material 1 (DOCX 2.05 MB) [file 18_2025_5849_MOESM1_ESM.docx]

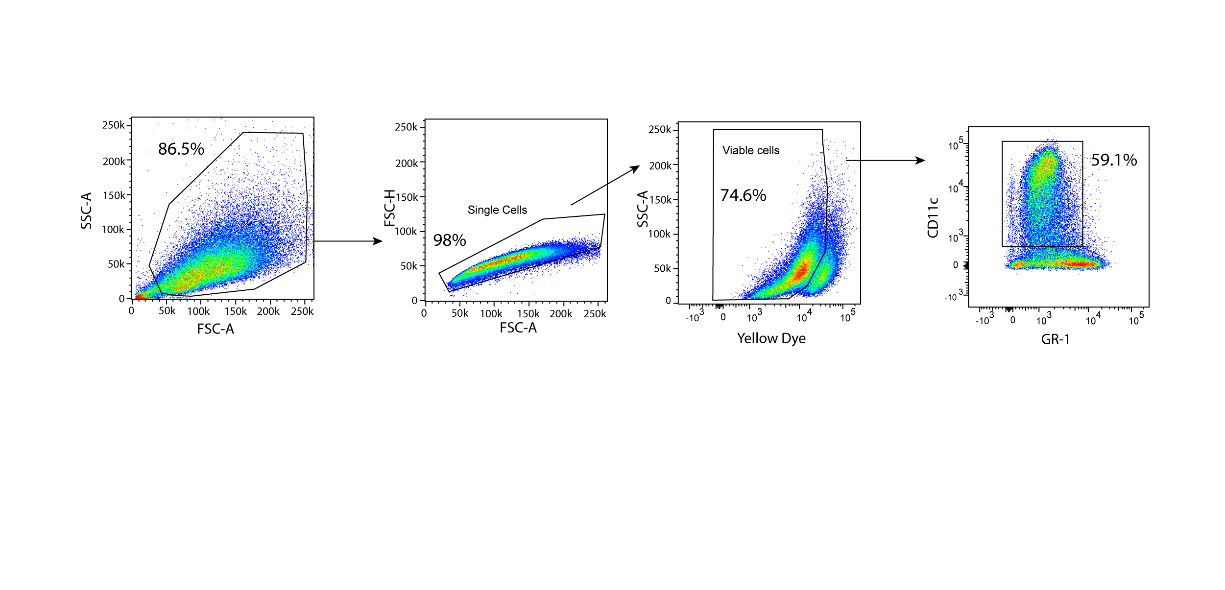


**Supplementary Figure 1. Flow cytometry gating strategy of BMDCs.** Gating strategy of mature BMDCs defined as live Gr1^-^ CD11c^+^ cells.


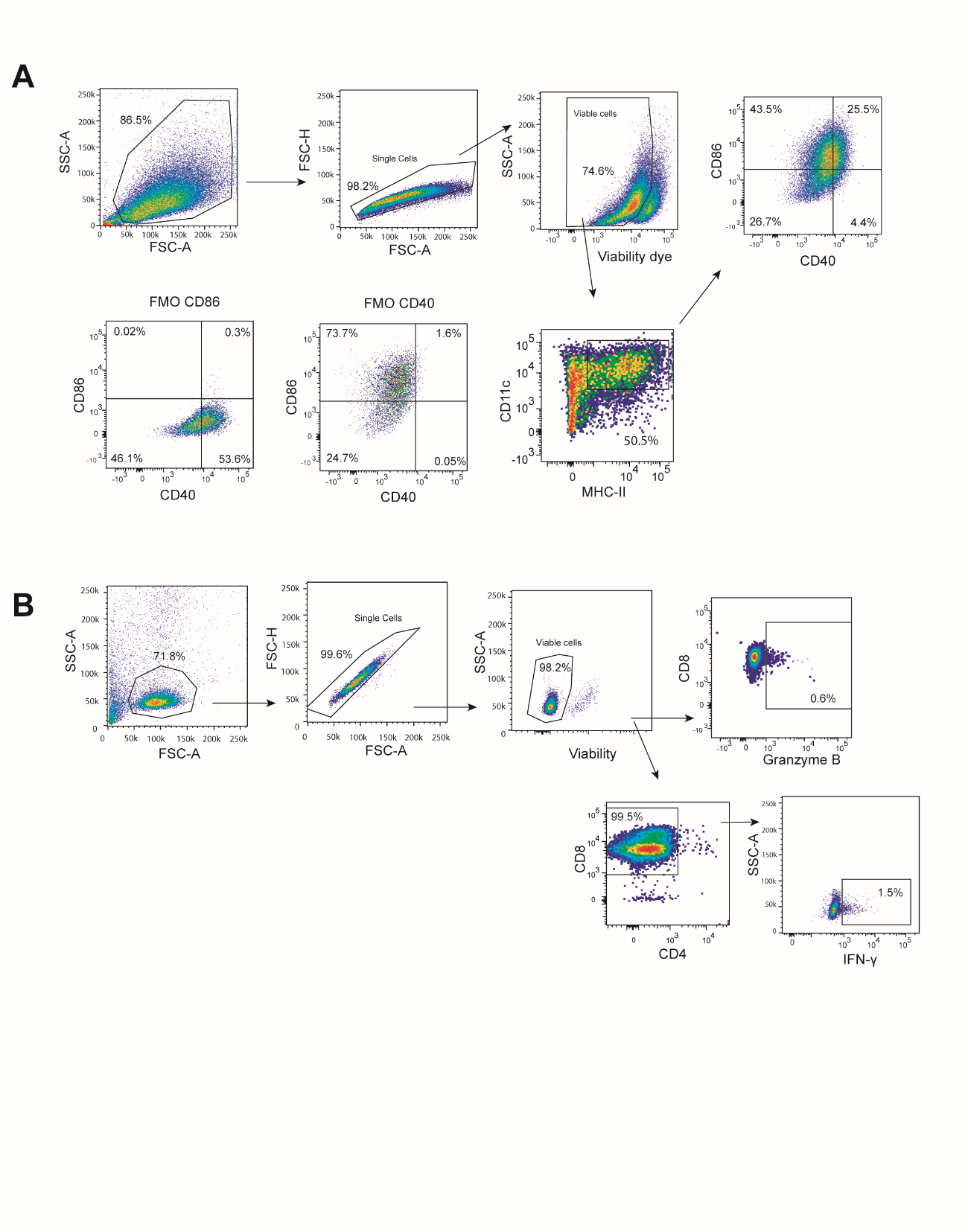


**Supplementary Figure 2. BMDC activation and modulation of T cell activation**. **(A)** Gating strategy of DC activation markers CD40 and CD86. **(B)** Gating strategy and frequencies of CD8+ T cells from OT-I mice expressing IFN-γ 24h after co-culture with BMDCs from WT or ISG15-KO with and without treatment with protein OVA and with OVA_257-264_ peptide.


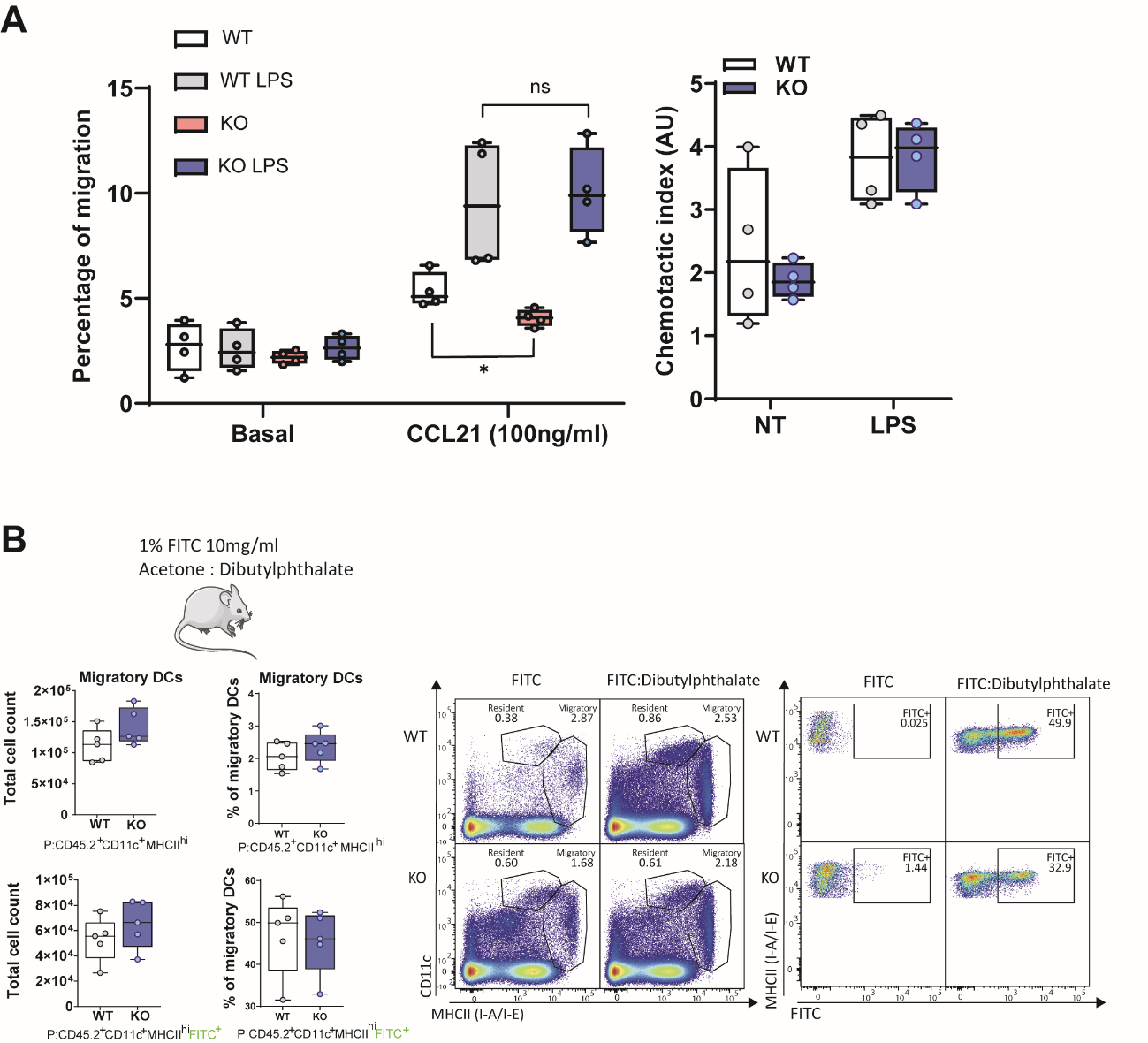


**Supplementary Figure 3. BMDC migration capacity. (A)** Percentage of migrated cells detected in the lower chamber (left panel) and the chemotactic index (right panel) in the presence or absence of CCL21 at a concentration of 100 ng/mL (n = 4 biological replicates, Student’s t test, *p < 0.05 and ns, not significant). NT: non-treated. **(B)** Schematic representation of skin painting model for DC migration study. Dot plot representation of the gaiting strategy for WT and KO migratory DCs at the aLN. Total count and frequencies of migratory DCs (upper panel) and those FITC+ (lower panel) at the aLN (n = 5 biological replicates, Student’s t test).


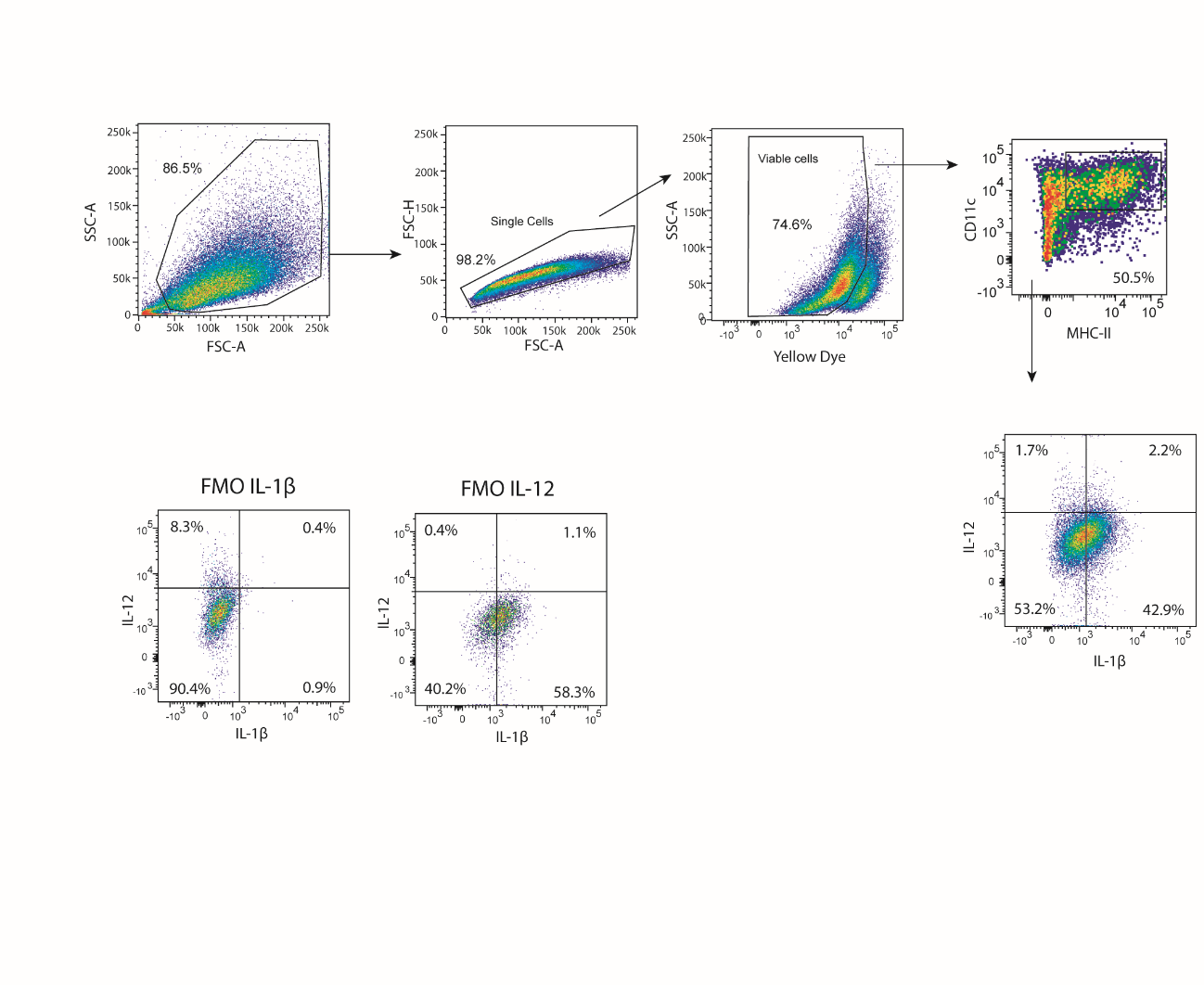


**Supplementary Figure 4. Gating strategy of intracellular stainings of IL-1β and IL-12 in a representative culture of BMDCs exposed to LPS.**


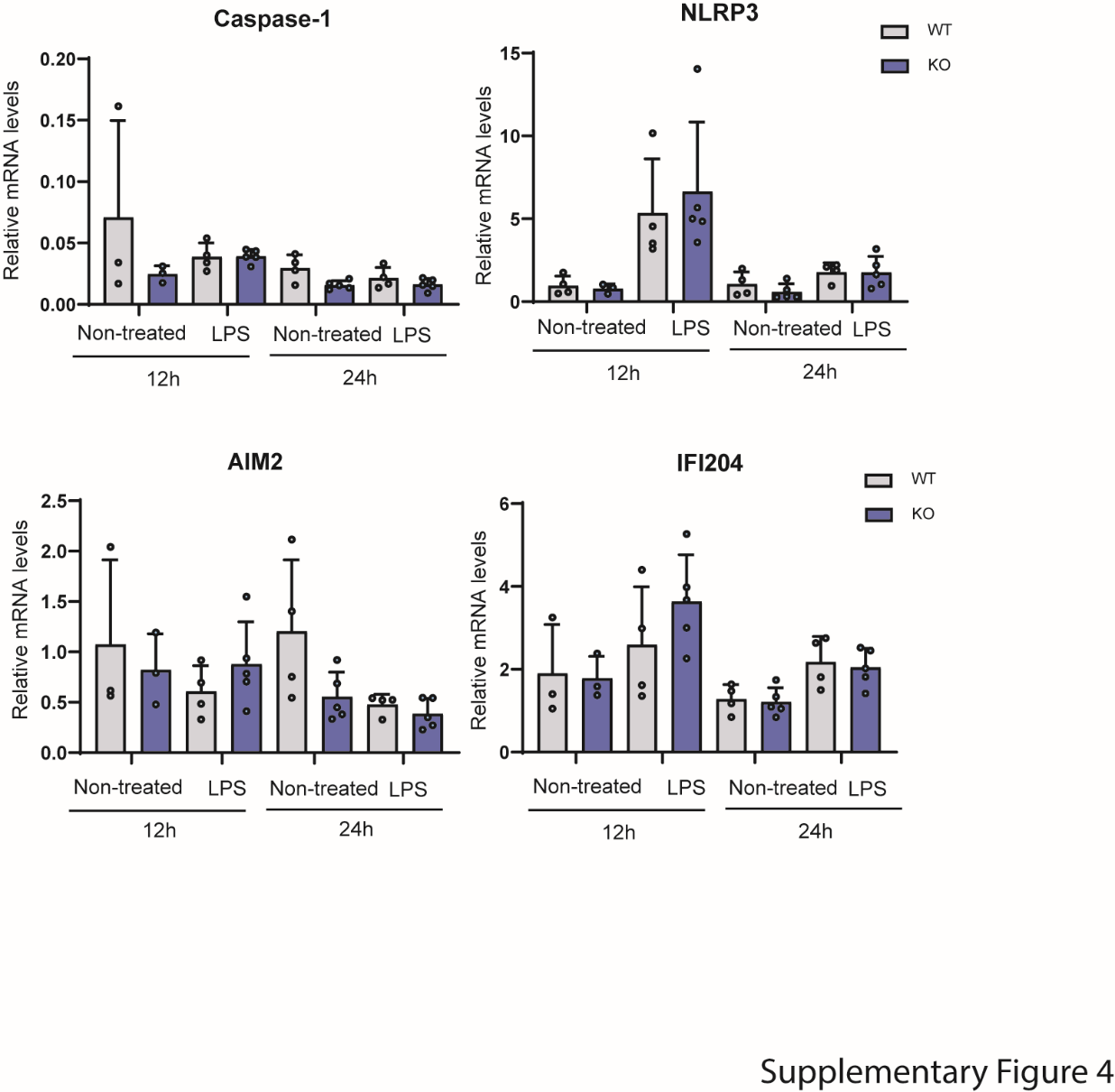


**Supplementary Figure 5. Expression of inflammasome components in ISG15-KO BMDCs.** Levels of mRNA by RT-qPCR were assessed 12h and 24h after treatment with LPS. N= 5 biological replicates, Mann-Whitney test.

| **Supplementary Table 1: Antibodies and other reagents** | | | |
| --- | --- | --- | --- |
| **Reagent** | **Manufacturer** | **Catalogue** | **Use** |
| **Flow cytometry** | | | |
| CD11c – BV421 | BD Horizon | 565452 | 1:200 FC |
| CD11c – PE | BD Pharmingen | 557401 | 1:200 FC |
| CD11c-PE-Cy7 | Tonbo Biosciences | 60-0114 | 1:200 FC |
| CD16/CD32 (Fc block) | Tonbo Biosciences | 70-0161 | 1:200 FC |
| CD16/CD32 (Fc block) | BD Biosciences | 553142 | 1:200 |
| CD3 – BV421 | BD Horizon | 562600 | 1:200 FC |
| CD4 – APC | Tonbo Biosciences | 20-0042 | 1:200 FC |
| CD40 – PerCPCy5.5 | BioLegend | 124624 | 1:200 FC |
| CD45 – Biotin | Hybridoma | NA | 1:100 IF |
| CD86 – PE | Tonbo Biosciences | 50-0862 | 1:200 FC |
| CD8α – APCFire750 | BioLegend | 100766 | 1:200 FC |
| CellTrace Violet Cell Proliferation Kit | Invitrogen | C34557 | 1:1000 FC |
| FoxP3 fix/ permeabilization kit | eBioscience | 00-5523 | NA |
| Granzyme B-PE-Cy7 | eBioscience | 25-8898-82 | 1:200 |
| IFN-γ-APC | Tonbo Biosciences | 20-7311-U100 | 1:200 |
| IFN-γ-FITC | Tonbo Biosciences | 37-7311-U100 | 1:200 |
| TNF-α-PE | BioLegend | 506306 | 1:100 |
| IL-1β-FITC | Invitrogen | 11-7114-82 | 1:100 |
| IL-12 (p40/p70)-APC | BD Biosciences | 554480 | 1:100 |
| GR-1 – V450 | Tonbo Biosciences | 75-5931 | 1:200 |
| LIVE/DEAD® Fixable Yellow Dead Cell Stain | Invitrogen | L34968 | 1:1000 |
| Ly-6C – PECy7 | BioLegend | 128017 | 1:200 |
| Ly-6G – PerCPCy5.5 | Tonbo Biosciences | 65-1276 | 1:200 |
| MHCII – FITC | BD Pharmingen | 553623 | 1:400 |
| MHCII – APC | eBioscience | 17-5321-81 | 1:400 |
| MHCII – APC | Invitrogen | 17-5321-81 | 1:300 |
| MHCII – APCFire750 | BioLegend | 107652 | 1:400 |
| UltraComp eBeads™ | Invitrogen | 01-2222-41 | - |
| **Western blot** | | | |
| Caspase-1 (mouse) | Invitrogen | 14-9832-82 | 1:500 |
| ISG15 antibody | Proteintech | 15981-1-ap | 1:500 |
| Vimentin | Sigma-Aldrich | V5255 | 1:500 |
| p-p65 | Cell Signaling | 3033S | 1:1000 |
| NLRP3 | Cell Signaling | 15101S | 1:1000 |
| p150 | BD | 610474 | 1:1000 |
| Goat α-mouse – HRP | Invitrogen | 31446 | 1:5000 |
| Goat α-rabbit – HRP | Invitrogen | 31460 | 1:5000 |
| Goat α-rat – HRP | Sigma-Aldrich | AP136P | 1:5000 |
| **TLR agonists** |  |  |  |
| Imiquimod (IMQ) | InvivoGen | tlrl-imqs | 1 µg/mL |
| CpG ODN2395 | InvivoGen | tlrl-2395 | 5 µg/mL |
| Lipopolysaccharide (LPS) | Sigma-Aldrich | L2630 | 1 µg/mL |
| Palmitoyl-2-CysSerLys-4 (Pam2CSK4) | InvivoGen | tlrl-pm2s | 10 µg/mL |
| Palmitoyl-3-CysSerLys-4 (Pam3CSK4) | InvivoGen | tlrl-pms | 10 µg/mL |
| Polyinosinic-polycytidylic acid (pI:C, HMW) | InvivoGen | tlrl-pic | 20 µg/mL |
| **Other reagents** | | | |
|  |  |  |  |
| Hu-IFN-$\alpha$A/D[Bg/II] | Pbl | 11200-2 | 100 U/mL |
| DNAseI | Roche | 10104159001 | 100 µg/mL |
| Fluorescein isothiocyanate (FITC) isomer I | Sigma-Aldrich | F7250-250MG | 1% |
| Mouse GM-CSF | PeproTech | 315-02 | 20 ng/mL |
| OT-Ip (OVA_257-264_): SIINFEKL | GenScript | NA | 1 µg/mL |
| OT-IIp (OVA_323-339_): ISQAVHAAHAEINEAGR | GenScript | NA | 5 µg/mL |
| Mouse CCL21 (Exodus-2) | Peprotech | 250-13 | 100 ng/mL |
| rmIL-12 | R&D Systems | 419-ML-010 | 20 ng/mL |
| rmISG15 | LSBio | LS-G26127-50 | 20 ng/mL |
| Anti-ISG15 polyclonal antibody | Cell Signaling | 2743S | 200 ng/mL |
| Isotype control antibody | BioLegend | 910801 | 200 ng/mL |

**Supplementary Table 1.** List of antibodies and other reagents.

| **Supplementary Table 2. Specific gene cDNA primers** | | | |
| --- | --- | --- | --- |
| **GENE** | **ID** | **Forward sequence (5'->3')** | **Reverse sequence (5'->3')** |
| *Il-12 p35* | 16159 | CTGTGCCTTGGTAGCATCTATG | GCAGAGTCTCGCCATTATGATTC |
| *Il-1β* | 16176 | AAAGACGGCACACCCACCCTGC | TGTCCTGACCACTGTTGTTTCCCAG |
| *Isg15* | 100038882 | CTAGAGCTAGAGCCTGCAG | AGTTAGTCACGGACACCAG |
| *Ubc* | NA | GCCCAGTGTTACCACCAAGA | CCCATCACACCCAAGAACA |
| *Hprt* | NA | GCAGTACAGCCCCAAAATGG | GGTCCTTTTCACCAGCAAGC |

**Supplementary Table 2.** List of qPCR primers.

| **Supplementary Table 3. ELISA** | | |
| --- | --- | --- |
| **Cytokine** | **Manufacturer** | **Catalogue No** |
| IL-1β | Invitrogen | 88-7013A-88 |
| IL-12p70 | Invitrogen | 88-7121-88 |

**Supplementary Table 3.** List of cytokine detection kits used in ELISA assays.
